# Sequencing 5‐Hydroxymethyluracil at Single‐Base Resolution

**DOI:** 10.1002/anie.201804046

**Published:** 2018-07-04

**Authors:** Fumiko Kawasaki, Sergio Martínez Cuesta, Dario Beraldi, Areeb Mahtey, Robyn E. Hardisty, Mark Carrington, Shankar Balasubramanian

**Affiliations:** ^1^ Department of Chemistry University of Cambridge Lensfield Road Cambridge CB2 1EW UK; ^2^ Cancer Research UK, Cambridge Institute Li Ka Shing Centre Robinson Way Cambridge CB2 0RE UK; ^3^ Department of Biochemistry University of Cambridge Hopkins Building Tennis Court Road Cambridge CB2 1QW UK; ^4^ School of Clinical Medicine University of Cambridge Cambridge CB2 0SP UK

**Keywords:** 5-formyluracil, 5-hydroxymethyluracil, DNA, epigenetics, thymine modifications

## Abstract

5‐hydroxymethyluracil (5hmU) is formed through oxidation of thymine both enzymatically and non‐enzymatically in various biological systems. Although 5hmU has been reported to affect biological processes such as protein–DNA interactions, the consequences of 5hmU formation in genomes have not been yet fully explored. Herein, we report a method to sequence 5hmU at single‐base resolution. We employ chemical oxidation to transform 5hmU to 5‐formyluracil (5fU), followed by the polymerase extension to induce T‐to‐C base changes owing to the inherent ability of 5fU to form 5fU:G base pairing. In combination with the Illumina next generation sequencing technology, we developed polymerase chain reaction (PCR) conditions to amplify the T‐to‐C base changes and demonstrate the method in three different synthetic oligonucleotide models as well as part of the genome of a 5hmU‐rich eukaryotic pathogen. Our method has the potential capability to map 5hmU in genomic DNA and thus will contribute to promote the understanding of this modified base.

DNA‐base modifications can profoundly influence biology and a number of modified bases have been identified in the genomes of a variety of organisms.^1^ 5‐hydroxymethyluracil (5hmU) is produced through oxidation of thymine both enzymatically and non‐enzymatically^2^ and can influence the binding of proteins to DNA.2b, 3 It has also been suggested that 5hmU might lead to genomic instability as it can be removed by DNA repair enzymes to create potentially mutagenic lesions^4^ and affect the stability of DNA duplexes.^5^ When incorporated at some promoter sites, 5hmU has been shown to affect transcription by bacterial RNAP, therefore it may have a significant effect on microbial biology.^6^ In mammals, reported levels of 5hmU vary by cell‐ and tissue types.2b, 7 Increased levels of 5hmU autoantibodies have been reported in cancer cases,^8^ and blood 5hmU mononucleoside levels have been studied as a marker of cancer risks and invasiveness.^9^ We previously reported a method to map 5hmU at moderate resolution by chemical enrichment of 5hmU‐containing DNA fragments followed by sequencing.^10^ A method for single‐base sequencing of 5hmU would enable the identification of individual modification sites in genomes. A single‐molecule real‐time (SMRT) sequencing approach could in principle be applied to map 5hmU at single‐base resolution, however the intrinsic signature signal for 5hmU is rather weak unless the base is further modified.^11^ Mapping 5hmU at single‐base resolution is a worthy challenge that could transform genome‐wide analysis of 5hmU. Herein, we describe a chemical approach for single base‐resolution sequencing of 5hmU and demonstrate its utility in various sequence contexts.

The conceptual basis for sequencing 5hmU involves chemical oxidation of 5hmU to 5fU, which ionizes under mild alkaline pH owing to the electron‐withdrawing exocyclic aldehyde (p*K*_a_ at N3=8.1 for 5fU vs. 9.3 for 5hmU).^12^ The ionized form of 5fU can base‐pair with G (Figure 1), causing a T‐to‐C base change that marks the original 5hmU sites. The oxidation of 5hmU to 5fU was carried out using KRuO_4_.^13^ The T‐to‐C change is then established during a polymerase‐dependent single extension, which is subsequently amplified by PCR. To rule out T‐to‐C changes that arise for reasons other than 5hmU (for example, pre‐existing mutations, non‐5hmU DNA damage), sequencing is compared to a “no‐oxidation” control in which there has been no conversion of 5hmU to 5fU.

**Figure 1:**
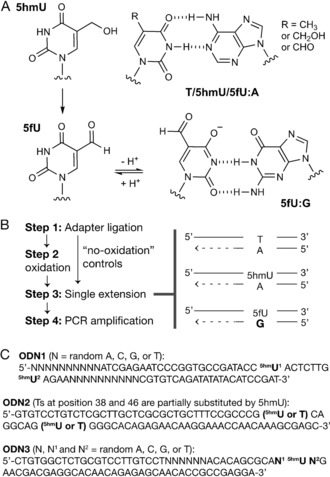
Structure of thymine‐base modifications and experimental designs. A) Canonical T:A, 5hmU:A, and 5fU:A base pairs and the 5fU:G base pair. B) The workflow to sequence 5hmU. C) Sequences of the oligonucleotide models (ODN).

As proof of concept, we first employed a synthetic oligonucleotide with two 5hmUs at defined positions (ODN1), and the base readout profiles at each 5hmU site as well as proximal non‐modified thymine sites were quantified (Table 1 and the Supporting Information, Tables S1 and S2). Sequencing was performed on an Illumina next generation sequencing (NGS) platform (a schematic summary of the sequencing data analysis is shown in the Supporting Information, Figure S1). Under optimised conditions, the proportion of total sequencing reads generating a “C” signal, at the 5hmU‐modified sites was high (“%C”=39 % and 30 %, Table 1 and Table S1) compared to unmodified T sites (1.4 %, Table 1; Wilcoxon rank‐sum test, *p*‐value=0.003 for both 5hmU sites). In the control experiment in which no oxidation of the DNA was carried out (that is, 5hmU is not converted to 5fU) the proportion of sequencing reads exhibiting a “C” signal at 5hmU‐modified sites were low (2.2 and 2.7 %, Table 1) and comparable to the levels of unmodified T (that is, 1.2 %, Table 1) (Wilcoxon rank‐sum test, *p*‐value >0.01, for both 5hmU sites). Thus, individual 5hmU sites could be resolved from unmodified T and detected by sequencing. We found that the T‐to‐C percentage change depends on the concentration of dATP during single‐extension PCR. A 500‐fold decrease in concentration of dATP compared to other nucleotide triphosphates was optimal (Table 1 and Table S1).

**Table 1:** Sequencing readout for 5hmU‐modified ODN1.

Protocol^[a]^	base	%T^[b]^	%C^[b]^	%other^[b]^
Steps 1–4	5hmU^1^	50.4±3.0	39.4±3.4	9.9±0.4
	5hmU^2^	65.1±1.4	30.3±1.5	4.6±0.3
	T^[c]^	98.2±0.3	1.4±0.3	<1
Steps 1, 3, and 4 (no‐oxidation control)	5hmU^1^	97.3±2.3	2.2±1.0	<1
	5hmU^2^	95.9±1.1	2.7±0.1	1.3±1.2
	T^[c]^	98.3±1.1	1.2±1.0	<1

[a] Steps as shown in Figure 1. Step 3 was carried out at 37 °C with 10 mm MgSO_4_ and dNTP mix (final concentrations: 250 μm for dCTP, dGTP, and TTP and 500 nm for dATP) using Bst DNA Polymerase, Large Fragment. See Supporting Information for details. [b] The proportion of reads giving T, C, or other signal (that is, A, G, insertion, and deletion) at the 5hmU‐modified sites over all reads. Mean±SD values of technical triplicates (at least two data out of three were obtained in coverage depth of greater than 1000×) are shown. [c] Mean values for seven proximal Ts.

The strength of the T‐to‐C signal change for 5fU depended on the choice of polymerase (Table S2), and we chose Bst DNA Polymerase, Large Fragment (obtained from New England Biolabs) for further study owing to its capability to induce T‐to‐C signal change at 5hmU sites without introducing noise at unmodified sites. When using a separate ODN model modified with varying levels of 5hmU at two defined positions (0–26 %, ODN2), the strength of the “C” signal at 5hmU (percent C counts over the sum of C and T counts, %C/(C+T)) increased linearly with the level of 5hmU (Figure S2 a). At a sequencing coverage depth of 100× in ODN2, 5hmU was detectable down to an incorporation level of 15 % (fold‐change of “C” signal compared to the no‐oxidation control, Figure S2b, data available at https://github.com/sblab-bioinformatics/5hmUseq).

To investigate potential sequence context bias in our approach, we prepared the oligonucleotide model ODN3, with a randomised base flanking each side of a single 5hmU site, therefore representing the 16 possible trinucleotide sequence contexts (N^1^‐5hmU‐N^2^, N^1^ and N^2^=A or T or G or C). While we observed some context‐dependent variability in the %C/(C+T) signal at the 5hmU‐modified sites, the calling of 5hmU relative to no‐oxidation control was clear and unambiguous in all cases (Figure 2), indicating that the method is suitable for detecting 5hmU in all trinucleotide sequence contexts.

**Figure 2:**
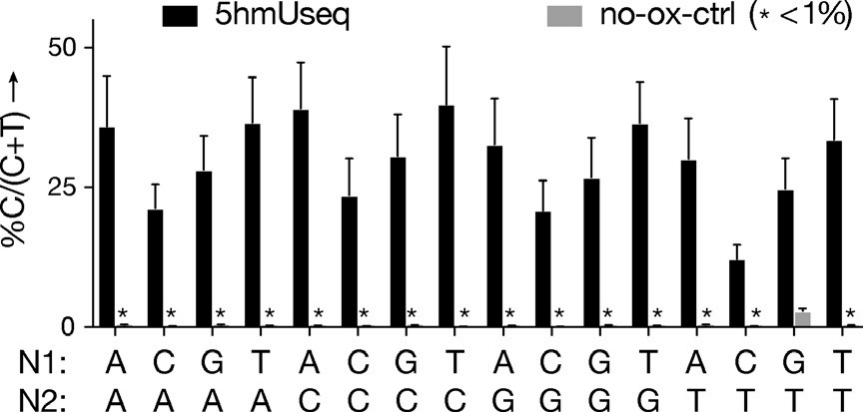
C signal in synthetic ODN3. The proportion of reads giving “C” signal over the total sum of C+T reads in N1‐5hmU‐N2 trinucleotide contexts in ODN3.

We then applied the method to map 5hmU in the genome of the eukaryotic pathogen *Trypanosoma brucei* (Figure 3).^14^ We mapped 5hmU on chromosome 2, and observed 161 Ts with significant 5hmU signal (0.02 % of all Ts on the chromosome), as defined using a FDR threshold (against “no‐oxidation” control) <0.1.^15^, ^16^ As determined using a simulated random distribution, these sites showed significant (*p*‐value=0.0019) overlap with 5hmU regions obtained using our previously reported chemical enrichment strategy (for details see Supporting Information).^10^, ^13^, ^16^

**Figure 3:**
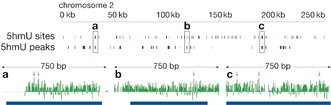
5hmU signal in chromosome 2 of *Trypanosoma brucei*. Ts with significant (FDR<0.1) single‐base resolution 5hmU sites and also regions of 5hmU peaks obtained using the previously reported chemical enrichment‐based method (top panel). Magnified view of three loci with 5hmU signal after normalization with the no‐oxidation controls (bottom panel). Arrows indicate the significant sites (FDR<0.1) ^[15]^ and bottom bars are 5hmU regions from enrichment mapping.

In conclusion, we have demonstrated a chemical method to detect and sequence 5hmU at single‐base resolution. We further envisage the method could be extended to detect 5fU by removing the oxidation step and normalising T‐to‐C conversion relative to conditions insensitive to 5fU such as libraries prepared without the single‐extension step.

## Experimental Section

Sequencing experiments were carried out on a Mi‐Seq instrument using Miseq reagent kit v3 (Illumina). Quantification of 5hmU by LC‐MS^2^ analysis was carried out on a Q Exactive™ Hybrid Quadrupole‐Orbitrap Mass Spectrometer (Thermo Scientific) equipped with a nanospray ionization source, coupled to an Ultimate RSLCnano LC system (Dionex). Detailed experimental protocols and commercial sources of reagents are included in the Supporting Information (PDF). All sequencing data have been deposited in the ArrayExpress database at EMBL‐EBI (https://www.ebi.ac.uk/arrayexpress/) under accession number E‐MTAB‐6456. All the code developed for the data analysis has been released in the manuscript's GitHub page (https://github.com/sblab-bioinformatics/5hmUseq).

## Conflict of interest

The authors declare no conflict of interest.

## Supporting information

As a service to our authors and readers, this journal provides supporting information supplied by the authors. Such materials are peer reviewed and may be re‐organized for online delivery, but are not copy‐edited or typeset. Technical support issues arising from supporting information (other than missing files) should be addressed to the authors.

Supplementary
