# Supplementary material for: Sequencing 5‐Hydroxymethyluracil at Single‐Base Resolution
Source: Angew Chem Int Ed Engl. 2018 Jul 4;57(31):9694–6. doi: 10.1002/anie.201804046 (PMC6100112; doi:10.1002/anie.201804046)
Supplement: Supplementary file 1 — Supplementary [file ANIE-57-9694-s001.pdf]

Angewandte Chemie
Eine Zeitschrift der Gesellschaft Deutscher Chemiker

Supporting Information

Sequencing 5-Hydroxymethyluracil at Single-Base Resolution

*Fumiko Kawasaki, Sergio Martínez Cuesta, Dario Beraldi, Areeb Mahtey, Robyn E. Hardisty, Mark Carrington, and Shankar Balasubramanian**

anie_201804046_sm_miscellaneous_information.pdf

Supporting Information 2016

Table of Contents

Experimental procedure

Protocol 1: Sequences and preparation of the model oligonucleotides (ODNs) (Page 2)

Protocol 2: Detection of 5hmU at single base resolution (Pages 2−4)

Protocol 3: Quantification of 5hmU levels for ODN2 (Page 4)

Protocol 4: Data access and analysis (Page 5)

Figure S1. Scheme depicting the steps to obtain %C/(C+T) signal, fold-change and significance for the difference between oxidation and no-oxidation in the synthetic ODN and genomic DNA libraries (Page 6)

Figure S2. C signal in ODN2 (Page 6)

Table S1. T-to-C signal change at each 5hmU site as well as proximal Ts by different conditions (Page 7)

Table S2. T-to-C signal change at each 5hmU site as well as proximal Ts by different enzymes (Page 7)

References and Author contributions (Page 8)

Appendix: Individual values used for Table 1, Table S1 and S2. (Page 9)

Experimental Procedures

Protocol 1: Sequences and preparation of the model oligonucleotides (ODN)

ODN 1: The model ODN shown in Table 1, Table S1–S2.
NNNNNNNNNNNATCGAGAATCCCGGTGCCGATACC**5hmU**[1]ACTCTTG**5hmU**[2]AGAANNNNNNNNNNNCGTGTCAGATATATACATCCGAT (N: random ATGC)
* Random sequences were used to identify unique reads in the sample to rule out potential PCR artefacts. Only sequencing reads from unique molecules, which were selected based on two randomized identifier sequences, were counted.
Sequence of the primer for dsDNA preparation: 5'-ATCGGATGTATATATCTGACACG-3'

A-1. Template ODNs were purchased from ATDbio. The following mixture was incubated at 95 ºC for 3 min then slowly cooled (–0.2 ºC /sec) to 37 ºC.

Reagents	µL
Template DNA (100 µM)	1
The primer for dsDNA preparation (100 µM)	5
NEB 2 buffer (New England Biolabs)	25
dNTP mix (10 mM each)	10
water	207
Total	248

Then Klenow Fragment (3'→5' exo-) (2 µL, NEW ENGLAND Biolabs) was added and the mixture was incubated at 37 ºC for 30 min. The dsDNA was purified by DNA Clean & Concentrator™-5 (Zymo Research).

ODN 2: The model ODN used for the experiment shown in Figure S1.
Template used for ODN2 preparation: GCTCGCTTTGTTGGTTTCCTTGTTCTCTGTGCCCACTGCCTGACGGGCGGAAAGCAGCGCGAGCAAGCGAGACAGGACAC (purchased from Life Technologies)
The primer for ODN2 preparation: 5'-GTGTCCTGTCTCGCTTGCTCGCGCTGCTTT-3'(purchased from Life Technologies)
The double-stranded ODN2 was prepared as in protocol A-1 except that TTP (10 mM) was substituted by a mixture of TTP and 5-hydroxymethyl-2'-deoxyuridine-5'-triphosphate (5hmdUTP, Trilink biotechnologies) in a ratio of 0:1, 1:1, 3:1, 7:1, 15:1, 1:0 to obtain fully, or partially 5hmU modified ODNs. 5hmU incorporation was estimated by mass spectrometry (see Protocol 3).

ODN 3: The model ODN used for the experiment shown in Figure 2.
CTGTGGCTCTGCGTCCTTGTCCTNNNNNNACACAGCGCAN5hmUNGAACGACGAGGCACAACAGAGAGCAACACCGCCGAGGA (N: random A or T or G or C)
* Pos 24-pos 29 were used to identify unique reads in the sample to eliminate potential PCR artefacts. Only sequencing reads from unique molecules, which were selected based the randomized identifier sequences, were counted.
Sequence of the primer for dsDNA preparation: 5'-TCCTCGGCGGTGTTGCTCTCTGTTGTGCCT-3'
The double-stranded ODN 3 was prepared as in protocol A-1.

Protocol 2: Detection of 5hmU at single-base resolution
Library preparation
A. Ligation
End prep and ligation were performed using NEBnext Ultra II kit (NEW ENGLAND Biolabs), following the manufacturer's directions. Adapter DNA was prepared as follows:
*Adapter stock solution
5'-MeO-GAATGATACGGCGACCACCGAGATCTACACTCTTTCCCTACACGACGCTCTTCCGATCT (adapter ODN 1)
3'-TCTGCACACGAGAAGGCTAGp (adapter ODN 2)

Adapter ODNs were obtained from Eurogentec (adapter ODN1) and Life Technologies (adapter ODN2). Adapter ODNs (10 µM each) were annealed in 10 mM Tris-HCl (pH 7.4) and NaCl (50 mM) at 95 ºC for 10 min then slowly cooled (–0.2 ºC /sec) to 4 ºC.

After the ligation reaction, the solution was diluted to 100 µL with water, and purified with AMPure® XP Beads (Beckman Coulter). 80% acetonitrile (1 mL) was used at each wash step instead of the standard wash solution (i.e. 80% ethanol). DNA was eluted with ultrapure water prepared by Merck Millipore Synergy UV system. An aliquot of the DNA sample (ca. 10 µL) was used for the no-oxidation control library (proceeded to step C without step D).

B. Oxidation of 5hmU to 5fU
Step B was not carried out for "no-oxidation" controls.

B-1. DNA sample from step A (up to 20–25 µL) was loaded on Bio-Gel P-6 in SSC Buffer (P6 column, Bio Rad), which was pre-washed with ultrapure water (5 times, 500 µL) prior to use.

B-2. The following mixture was incubated at 37 ºC for 30 min.

Reagents	µL
DNA sample from step B-1	x
Water	45.5-x
1 M NaOH	2.5
Total	48

B-3. The following mixture was incubated at 25 ºC for 30 min. The oxidant solution provided in the TrueMethyl™ kit (Cambridge Epigenetix) was diluted 10-fold with ultrapure water prior to use.

Reagents	µL
Mixture from step B-2	48
Diluted oxidant solution in the TrueMethyl™ kit	2
Total	50

After incubation, another portion of the diluted oxidant solution (2 µL) was added and the mixture was further incubated at 25 ºC for 30 min. Then the mixture was purified on Bio-Gel P-6 in SSC Buffer (P6 column, Bio Rad), which was pre-washed with ultrapure water (3 times, 500 µL) prior to use.
Alternatively, the oxidation reaction can be performed using a solution of $KRuO_4$ in aqueous NaOH. See reference 13 in the manuscript.

C. Single extension
Step C was not carried out for “no-extension” controls.

C-1. The following composition was used. For libraries prepared in other single extension procedures, the compositions were adjusted accordingly.

Reagents	µL
DNA sample from step B (or A for “no-oxidation” controls)	x
Water	13.5-x
100 mM $MgSO_4$	1.6
NEBNext Index Primer for Illumina (NEW ENGLAND Biolabs)	2
ThermoPol® Reaction Buffer (x10) (NEW ENGLAND Biolabs)	2
dNTP mix (to make the final concentration given in Table 1, S1–2)	0.5
Total	19.6

C-2. The following mixture was incubated at 37 ºC for 1 h.

Reagents	µL
Mixture from step C-1	
Bst DNA Polymerase, Large Fragment (NEW ENGLAND Biolabs)	0.4
Total	20

After the incubation, DNA was purified using the DNA Clean & Concentrator™-5 (Zymo Research) or GeneJet PCR purification kit (Thermo Fischer Scientific), and eluted with water.

D. PCR amplification, sample purification, and quantification
The following mixture was amplified by PCR. Primer sequences are shown below.

Reagents	µL
DNA sample from step C	10
Primer solution*	2.5
NEBNext® Ultra II Q5® Master Mix (x2) (NEW ENGLAND Biolabs)	12.5
Total	25

*Primer solution
Primer 1: 5’-AATGATACGGCGACCACCGAGATCTACACTCTTTCCCTACACGA-3’
Primer 2: 5’-CAAGCAGAAGACGGCATACGAGAT-3
Adapter oligonucleotides ODN were obtained from Life Technologies. The primer solution contains the primers (10 µM each) in water.

For the “no-extension” controls, PCR mixture was as follows.

Reagents	µL
DNA sample from step B	10
NEBNext Index Primer for Illumina (NEW ENGLAND Biolabs)	1.25

NEBNext Universal Primer for Illumina (NEW ENGLAND Biolabs)	1.25
NEBNext® Ultra II Q5® Master Mix (x2)	12.5
Total	25

The PCR protocol:

CYCLE STEP	TEMP	TIME	CYCLES
Initial Denaturation	98 °C	30 seconds	1
Denaturation Annealing/Extension	98 °C/65 °C	10 seconds/75 seconds	6–8
Final Extension	65 °C	5 minutes	1
Hold	4 °C		

After PCR, the solution was diluted to 100 µL with water, purified with AMPure® XP Beads, and eluted with water (22 µL). If any adapter/primer derived impurities were observed, the sample was diluted to 50 µL with water, purified with AMPure® XP Beads (50 µL) again, and eluted with water (22 µL). The library was quantified using KAPA Library Quantification Kit (KAPA Biosystems).

Protocol for *Trypanosoma brucei* (*Trypanosoma brucei brucei EATRO1125, blood stream form*) genomic DNA
Genomic DNA samples were fragmented to the average size of 200bp using an M220 Focused-ultrasonicator™ (Covaris). End prep and ligation were performed as in step A (page 3). An aliquot of the DNA sample obtained was used for the "no-oxidation" control library (proceeded to step C without step B). The adapter-ligated sample was subjected to oxidation as in step B (page 3). The single extension step was carried out as follows (step C').

C-1'. The following composition was used. For libraries prepared in other single extension procedures, the compositions were adjusted accordingly.

Reagents	µL
DNA sample from step B	x (typically 50-55 µL)
water	58-x
100 mM $MgSO_4$	6.4
NEBNext Index Primer for Illumina (NEW ENGLAND Biolabs)	4
ThermoPol® Reaction Buffer (x10) (NEW ENGLAND Biolabs)	8
dNTP mix (10 mM for dCTP, dGTP, and TTP; 20 µM for dATP)	2
Total	78.4

C-2'. The following mixture was incubated at 37 ºC for 1 h.

Reagents	µL
Mixture from step C-1	
Bst DNA Polymerase, Large Fragment (NEW ENGLAND Biolabs)	1.6
Total	80

After the incubation, DNA was purified using the DNA Clean & Concentrator™-5 (Zymo Research) and eluted with hot water (22 µL). The libraries were further amplified as in step D (Page 4), but in a 50 µL (x2) scale.

Protocol 3. Quantification of 5hmU levels for ODN2
DNA samples (ca. 1 µg) were treated with DNA Degradase Plus™ (0.1 U/µL, ZYMO RESEARCH). After incubation at 37 ºC for 4 h, the solution was filtered with Amicon Ultra-0.5 mL 10 K centrifugal filters (Merck Millipore) and subjected to LC-MS^2 analysis on a Q Exactive™ Hybrid Quadrupole-Orbitrap Mass Spectrometer (Thermo Scientific). This was equipped with a nanospray ionization source, coupled with an Ultimate 3000 RSLCnano HPLC system (Dionex) installed with a commercially sourced Hypercarb KAPPA column (30 mm x 0.18 mm ID, 5 μm particle size, Thermo Fisher) and connected with 2 x nanoviper connectors (75 μm x 150 mm). Samples were injected onto the column via the loading pump in 95:5 0.1% formic acid H_2O:MeCN with a flow rate of 2 µL min^{-1}. A valve switch to the NC pump followed after 5 min; the flow-rate was set to 1.5 uL min^{-1} and run with a gradient of 95:5 to 0:100 0.1% formic acid H_2O:MeCN with a run-time of 19 min. Parent ions were fragmented in positive ion mode with 10% normalised collision energy in parallel-reaction monitoring (PRM) mode. MS^2 resolution was 35,000 with an AGC target of $2e^5$, a maximum injection time of 100 ms and an isolation window of 1.0 m/z. Extracted ion chromatograms (±5ppm) were used for the detection and quantification. Quantification was performed using the XCalibur QuanBrowser software (Thermo Fisher) via internal calibration with stable isotope labelled (SIL) standards for dT and 5hmU.
For the quantification, calibration curves were generated by preparing a dilution series of the analytes (dT 4.5–2500 nM and 5hmU 0.2–125 nM), with each calibration point spiked with D_3-dT (25 nM) and $^{13}CD_2$-5hmU (2.5 nM) SILs as internal standards. The concentrations of the nucleoside standards were then plotted against the mass integration area ratio of nucleoside/SIL using the QuanBrowser software (Thermo Fisher). To each digested synthetic ODN sample was added an equivalent concentration of SIL standards to that of the calibration curves. The mass integration area ratio of nucleoside/SIL was then compared to the linear fit equation of calibration curves using QuanBrowser software (Thermo Fisher) to determine the nucleoside concentration. The obtained signals were normalised by ODN2 synthesized without dTTP (fully modified at position 35 and 42 with 5hmU).

Protocol 4. Data access and analysis
All the sequencing data have been deposited in the ArrayExpress database (https://www.ebi.ac.uk/arrayexpress/) at EMBL-EBI under accession number E-MTAB-6456 (https://www.ebi.ac.uk/arrayexpress/experiments/E-MTAB-6456).

All the code developed for the data analysis has been released in the manuscript's GitHub page (https://github.com/sblab-bioinformatics/5hmUseq). The base quality of reads was evaluated with FastQC v0.11.3. Low-quality bases were filtered and Illumina sequencing adaptors were trimmed from the 3' end of the read using cutadapt v1.12 [1] Formatting, analysis and statistical testing of the resulting tables was performed using python v2.7.12 [2] and R v3.3.2 [3] programming languages.

ODN 1 experiment (relevant to Table 1, Table S1, S2):
Only reads longer than 90bp after trimming and containing the adaptor were analysed. Reads were split into forward or reverse of the ODN 1 reference sequence and random barcodes were put into the read header and reads containing the same barcode were deduplicated. Resulting reads were aligned to the ODN 1 reference sequence using bwa mem v0.7.15-r1140 [4] with parameters –k 5 –T 10 –L 200 for sensitive alignment and nucleotide frequencies (A, C, G, T) were counted using pysamstats v1.0.1. [5] Output tables were finally combined.
To compare differences in the mean ranks of C signal in 5hmU (x2) and unmodified T (x7) sites, we used the Wilcoxon rank-sum test, an alternative of the Student's t-test, which does not require the assumption of normal distribution of values obtained from our experimental triplicates.[6] The 3 individual values for each 5hmU were compared against the 21 values (7x3) for each unmodified T.

ODN 2 experiment (relevant to Figure S1):
Filtered reads were aligned to the ODN 2 reference sequence using bwa mem v0.7.15-r1140[4] with parameters –k 5 –T 10 –L 200 for sensitive alignment. Samples simulating a coverage depth of 100x were obtained from the resulting bam files using bedtools v2.26.0 [7] and nucleotide frequencies (A, C, G, T) were counted using pysamstats v1.0.1. [5] Comparing the two groups of triplicates for the "oxidation" and "no oxidation" treatments was performed using edgeR v3.16.5.[8] Group dispersions were calculated to allow the fitting of a negative binomial generalised linear model (glm) to the C and T counts. The contrast "oxidation" - "no oxidation" was defined and likelihood ratio test was carried out. Fold changes (FC) were then computed for every T position, and false discovery rates (FDR) were estimated using the Benjamini-Hochberg method.

ODN 3 experiment (relevant to Figure 2):
The quality check and filtering of sequencing reads was performed as for the ODN 2 experiment. Filtered reads were matched to the forward or reverse of the ODN 3 reference sequence. All combinations of 6-mer random barcode sequences and the two nucleotides neighboring the 5hmU site were catalogued for the forward and reverse sequences separately. Frequencies of C and T counts at the 5hmU site for each combination of barcode and flanking dinucleotide were calculated by (a) using all sequences linked to the same barcode, (b) selecting one sequence at random from the group of sequences linked to the same barcode.

Mapping 5hmU sites and 5hmU regions in chromosome 2 of *Trypanosoma brucei* (relevant to Figure 3):
As 5hmU is read as C after conversion, the reference genome was prepared by appending two reference genomes where (a) all Ts are changed into Cs, and (b) all As are changed into Gs. Filtered single-end reads were also T to C converted and aligned to the converted reference using bwa mem v0.7.15-r1140[4] with default parameters. Alignments were cleaned to remove unmapped reads, not primary alignments and reads low alignment quality, chromosomes were renamed and their sequences fixed, with alignments merged, sorted and indexed using using samtools v1.3.1 [9] and in-house scripts (https://github.com/sblab-bioinformatics/5hmUseq). Nucleotide frequencies (Ts and Cs at T positions in the reference genome) were counted using pysamstats v1.0.1 [5] and bioawk v20110810. Filtering by counts, calculating %C/(C+T) signal and obtaining significant 5hmU sites was performed in the R v3.3.2 [3] programming language using the library limma v3.30.11 [10].

Filtered paired-end reads from the chemical enrichment-based method [11, 12] were aligned to the unconverted reference genome using bwa mem v0.7.15-r1140[4] with default parameters. Duplicate pairs were marked using Picard Tools [13] alignments were cleaned to remove unmapped reads, not primary alignments, duplicated reads and reads with low alignment quality, and indexed using samtools v1.3.1 [9]. Peaks were called using macs2 callpeak [14] with options "--nomodel --keep-dup all -p 0.00001 -g 30e6". Consensus peaks were obtained using bedtools v2.26.0 [7] and in-house scripts (https://github.com/sblab-bioinformatics/5hmUseq).

Single-base resolution 5hmU sites present in chromosome 2 and 5hmU regions obtained from the chemical enrichment-based method were extracted using standard unix tools. To explore the association between sites and regions, bedtools v2.26.0 [7] was used, and simulations were performed using the Genomic Association Tester GAT [15]. Genome tracks were visualised using the Integrative Genomics Viewer IGV v.2.4.10 [16].

1) Obtain sequencing reads and quality control

2) Align, deduplicate and count sequencing reads

**alignment references**	synthetic ODN libraries: 5hmU sites = T<br>genomic DNA libraries: all Ts = T and C

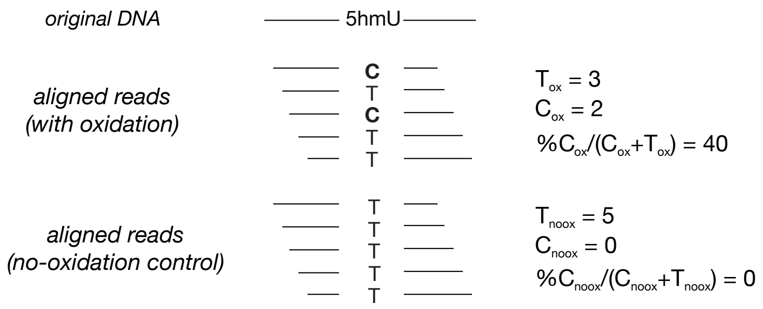

3) Fold-change calculation and statistics

**significance models**		
synthetic ODN libraries	negative binomial generalised linear model of counts	[(T_{ox}, C_{ox}) - (T_{noox}, C_{noox})]
genomic DNA libraries	logit transformation and linear model of conversions	[$\%C_{ox}/(C_{ox}+T_{ox})$ - $\%C_{noox}/(C_{noox}+T_{noox})$]

Figure S1. Scheme depicting the steps to obtain %C/(C+T) signals, fold-change and significance for the difference between oxidation and no-oxidation in the synthetic ODN and genomic DNA libraries.

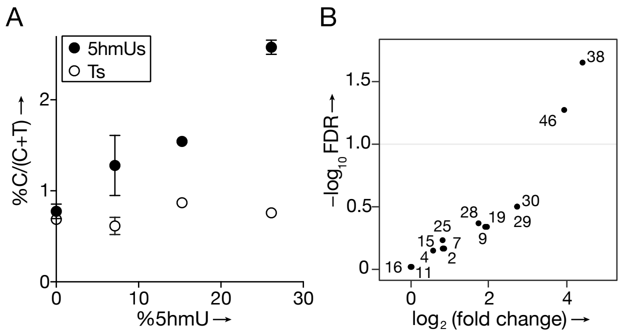

Figure S2. C signal in ODN2. (A) The percentage of reads giving C signal in ODN2. Mean 5hmU incorporation rates measured by mass spectrometry as well as mean percentage of C signals over the C+T total for two potential 5hmU incorporation sites are shown. As a control, mean percentage of C signals over the C+T total for all other unmodified T sites are shown. (B) A plot of fold change of C signal relative to the “no-oxidation” control (x-axis) and corresponding FDR-corrected p-values (y-axis) in ODN with 15% 5hmU incorporation. Data for each 5hmU-modified site (38 and 46) as well as other non-modified T bases (2, 4, 7, 9, 11, 15, 16, 19, 25, 28, 29, 30) were plotted with labels.

Table S1. T-to-C signal change at each 5hmU site as well as proximal Ts in ODN1 using different conditions

ODN1: 5'---ATCGAGAATCCCGGTGCCGAACC5hmU[1]ACTCTTG5hmU[2]AGAAT---3'

	5hmU[1] readout[a]			5hmU[2] readout[a]			Proximal Ts[a]		
Conditions in the single extension step[b]	%T	%C	%other	%T	%C	%other	%T	%C	%other
dATP concentration 500 nM	50.4±3.0[c]	39.4±3.4[c]	9.9±0.4[c]	65.1±1.4[c]	30.3±1.5[c]	4.6±0.3[c]	98.2±0.3[c]	1.4±0.3[c]	<1[c]
dATP concentration 5 µM	66.7	22.2	11.1	84.1	10.2	5.7	>99	<1	<1
No single extension step ("no-extension" control)	85.9	1.0	13.0	98.1	<1	1.2	>99	<1	<1

[a] The proportion of reads giving T, C, or other signal (i.e. A, G, insertion, and deletion) at the 5hmU-modified sites over all reads. Mean ± s.d. (for technical triplicates) or mean values (for technical duplicates) are shown (see Appendix for individual values). [b] Single extension was carried out at 37 °C in presence of 10 mM of $MgSO_4$, dNTP mix (final concentrations: 250 µM for dCTP, dGTP, TTP and dATP as indicated) and Bst DNA Polymerase Large Fragment (NEW ENGLAND Biolabs) in ThermoPol® Reaction Buffer (NEW ENGLAND Biolabs). [c] Values were obtained from Table 1 for comparison.

Table S2. T-to-C signal change at each 5hmU site as well as proximal Ts in ODN1 by different enzymes.

	5hmU[1] readout[a]			5hmU[2] readout[a]			Proximal Ts[a]		
Enzymes used for single extension[b]	%T	%C	%other	%T	%C	%other	%T	%C	%other
Klenow Large Fragment (3'→5' exo-) (NEW ENGLAND Biolabs)	43.9	44.4	11.8	53.8	39.0	7.2	95	4.1	<1
Deep Vent® (exo-) DNA Polymerase (NEW ENGLAND Biolabs)	53.5	33.1	13.4	61.9	31.4	6.7	98.2	<1	<1
Sulfolobus DNA Polymerase IV (NEW ENGLAND Biolabs)	92.8	<1	6.4	97.8	1.2	1.0	>99	<1	<1

[a] The proportion of reads giving T, C, or other signal (i.e. A, G, insertion, and deletion) at the 5hmU-modified sites over all reads. Mean values. of technical duplicates are shown (see Appendix for individual values). [b] Single extension was carried out at 37 °C in ThermoPol® Reaction Buffer with 10 mM $MgSO_4$ and dNTP mix (250 µM for dCTP, dGTP, TTP and 500 nM for dATP) using 0.4 µL of each enzyme on a 20 µL reaction scale.

References

[1] M. Martin, *EMBnet.journal* **2011**, *17*.

[2] Python Software Foundation. Python Language Reference, version 2.7. Available at http://www.python.org.

[3] Team, R. C. A language and environment for statistical computing. Available at https://www.R-project.org/

[4] H. Li, **2013**, arXiv:1303.3997.

[5] pysamstats: a fast Python and command-line utility for extracting simple statistics against genome positions based on sequence alignments from a SAM or BAM file. Available online at: https://github.com/alimanfoo/pysamstats

[6] M. J. Crawley, The R Book, John Wiley & Sons Ltd, 2007.

[7] A. R. Quinlan, I. M. Hall, *Bioinformatics* **2010**, *26*, 841-842.

[8] M. D. Robinson, D. J. McCarthy, G. K. Smyth, *Bioinformatics* **2010**, *26*, 139-140.

[9] H. Li, B. Handsaker, A. Wysoker, T. Fennell, J. Ruan, N. Homer, G. Marth, G. Abecasis, R. Durbin, 1000 Genome Project Data Processing Subgroup, *Bioinformatics* **2009**, *25*, 2078-2079.

[10] M. E. Ritchie, B. Phipson, D. Wu, Y. Hu, C. W. Law, W. Shi, G. K. Smyth, *Nucleic Acids Res.* **2015**, *43*, e47.

[11] R. E. Hardisty, F. Kawasaki, A. B. Sahakyan, S. Balasubramanian, *J Am Chem Soc.* **2015**, *137*, 9270-9272.

[12] F. Kawasaki, D. Beraldi, R. E. Hardisty, G. R. McInroy, P. van Delft, S. Balasubramanian, *Genome Biol.* **2017**, *30*, 23.

[13] Picard is a set of command line tools for manipulating high-throughput sequencing (HTS) data and formats such as SAM/BAM/CRAM and VCF. Available online at: http://broadinstitute.github.io/picard/

[14] Y. Zhang, T. Liu, C. A. Meyer, J. Eeckhoute, D. S. Johnson, B. E. Bernstein, C. Nusbaum, R. M. Myers, M. Brown, W. Li, X. S. Liu, *Genome Biol.* **2008**, *9*, R137.

[15] A. Heger, C. Webber, M. Goodson, C. P. Ponting, G. Lunter, *Bioinformatics* **2013**, *29*, 2046-2048.

[16] H. Thorvaldsdottir, J. T. Robinson, J. P. Mesirov, *Brief Bioinform.* **2013**, *14*, 178-192.

Author Contributions

FK and SB conceived the approach. FK planned and carried out the sequencing study; SMC and DB carried out data analysis; AM and REH carried out the mass spectrometry analysis. MC provided genome DNA samples and assisted the data interpretation. The manuscript was written by FK, SMC and SB with contributions from all authors. All authors have given approval to the final version of the manuscript.

Appendix. Individual values used for Table 1, Table S1 and S2.

%T

Table 1	Conditions for single extension PCR	library ID	T1	T2	T3	T4	T5	T6	T7	Average Ts	5hmU1	5hmU2
Steps 1–4	Bst DNA Polymerase, Large fragment,	fk386	100.0	97.7	96.4	96.8	97.4	97.7	99.1	97.9	53.7	64.1
	37 deg, ThermoPol® Reaction Buffer,	fk449	100.0	98.6	97.7	97.9	98.0	98.1	99.1	98.5	50.0	66.7
	10 mM MgSO4, dATP concentration	fk450	100.0	98.7	97.5	97.6	97.8	98.0	98.8	98.3	47.7	64.5
Steps 1, 3	Bst DNA Polymerase, Large fragment,	fk421	100.0	97.7	96.5	97.0	97.4	96.8	98.5	97.7	95.9	96.5
and 4 (“no-	37 deg, ThermoPol® Reaction Buffer,	fk422	100.0	97.7	96.4	96.7	97.3	96.7	98.4	97.6	96.0	96.7
oxidation”	10 mM MgSO4, dATP concentration	fk277	100.0	100.0	100.0	100.0	100.0	100.0	97.4	99.6	100.0	94.6
Table S1, S2												
Bst DNA Polymerase, Large fragment, 37 deg,		fk380	100.0	99.5	99.5	99.8	99.6	99.4	99.6	99.6	66.9	85.0
ThermoPol® Reaction Buffer , 10 mM MgSO4, dATP		fk381	100.0	99.6	99.5	99.5	99.6	99.5	99.7	99.6	66.6	83.3
"no-	No single extension step	fk457	100.0	99.7	99.8	99.8	99.6	99.6	99.6	99.7	86.1	98.0
extension"		fk458	100.0	99.8	99.7	99.7	99.6	99.6	99.7	99.7	85.8	98.2
Klenow Large Fragment (3'→5' exo), 37 deg,		fk419	100.0	94.3	92.6	95.4	95.1	95.0	94.1	95.2	45.6	57.5
ThermoPol® Reaction Buffer I, 10 mM MgSO4, dATP		fk420	100.0	92.2	89.4	93.6	95.6	95.2	97.4	94.8	42.1	50.1
Deep Vent® (exo-) DNA Polymerase , 37 deg,		fk423	100.0	98.0	96.8	98.0	97.5	97.3	98.6	98.0	51.3	59.5
ThermoPol® Reaction Buffer I, 10 mM MgSO4, dATP		fk424	100.0	99.0	98.0	98.4	97.7	97.7	98.5	98.5	55.7	64.3
Sulfolobus DNA Polymerase IV , 37 deg,		fk459	100.0	99.7	99.5	99.6	99.5	99.6	99.7	99.7	92.5	98.2
ThermoPol® Reaction Buffer I, 10 mM MgSO4, dATP		fk460	100.0	99.3	99.1	99.2	99.2	98.8	99.5	99.3	88.4	94.5

%others

Table 1	Conditions for single extension PCR	library ID	T1	T2	T3	T4	T5	T6	T7	Average Ts	5hmU1	5hmU2
Steps 1–4	Bst DNA Polymerase, Large fragment,	fk386	0.0	0.4	0.5	0.7	0.7	0.5	0.3	0.5	10.3	4.3
	37 deg, ThermoPol® Reaction Buffer,	fk449	0.0	0.3	0.5	0.6	0.7	0.5	0.3	0.4	9.7	4.6
	10 mM MgSO4, dATP concentration	fk450	0.0	0.2	0.5	0.5	0.7	0.6	0.3	0.4	9.6	4.9
Steps 1, 3	Bst DNA Polymerase, Large fragment,	fk421	0.0	0.4	0.5	0.5	0.4	1.2	0.8	0.5	0.8	0.7
and 4 (“no-	37 deg, ThermoPol® Reaction Buffer,	fk422	0.0	0.4	0.6	0.5	0.4	1.3	0.8	0.6	0.8	0.7
oxidation”	10 mM MgSO4, dATP concentration	fk277	0.0	0.0	0.0	0.0	0.0	0.0	2.6	0.4	0.0	2.7
Table S1, S2												
Bst DNA Polymerase, Large fragment, 37 deg,		fk380	0.0	0.4	0.3	0.1	0.3	0.5	0.3	0.3	12.2	5.6
ThermoPol® Reaction Buffer , 10 mM MgSO4, dATP		fk381	0.0	0.2	0.3	0.3	0.3	0.4	0.2	0.2	9.9	5.8
"no-	No single extension step	fk457	0.0	0.3	0.2	0.2	0.3	0.3	0.2	0.2	12.9	1.3
extension"		fk458	0.0	0.2	0.3	0.3	0.2	0.3	0.2	0.2	13.2	1.1
Klenow Large Fragment (3'→5' exo), 37 deg,		fk419	0.0	1.0	1.1	0.9	2.4	1.1	1.1	1.1	13.2	7.4
ThermoPol® Reaction Buffer I, 10 mM MgSO4, dATP		fk420	0.0	0.7	1.6	1.2	1.2	0.7	0.1	0.8	10.4	7.1
Deep Vent® (exo-) DNA Polymerase , 37 deg,		fk423	0.0	0.9	1.5	0.9	1.6	1.8	1.0	1.1	13.7	7.2
ThermoPol® Reaction Buffer I, 10 mM MgSO4, dATP		fk424	0.0	0.4	0.9	0.7	1.4	1.5	1.1	0.8	13.1	6.2
Sulfolobus DNA Polymerase IV , 37 deg,		fk459	0.0	0.3	0.5	0.3	0.4	0.3	0.2	0.3	6.6	0.8
ThermoPol® Reaction Buffer I, 10 mM MgSO4, dATP		fk460	0.0	0.4	0.2	0.7	0.4	0.6	0.2	0.4	6.0	2.1

%C

Table 1	Conditions for single extension PCR	library ID	T1	T2	T3	T4	T5	T6	T7	Average Ts	5hmU1	5hmU2
Steps 1–4	Bst DNA Polymerase, Large fragment,	fk386	0.0	1.9	3.0	2.5	1.9	1.8	0.6	1.7	36.0	31.6
	37 deg, ThermoPol® Reaction Buffer,	fk449	0.0	1.1	1.8	1.6	1.3	1.4	0.7	1.1	40.3	28.7
	10 mM MgSO4, dATP concentration	fk450	0.0	1.1	2.0	1.9	1.5	1.4	0.8	1.3	42.7	30.6
Steps 1, 3	Bst DNA Polymerase, Large fragment,	fk421	0.0	2.0	3.0	2.6	2.2	2.0	0.7	1.8	3.3	2.8
and 4 (“no-	37 deg, ThermoPol® Reaction Buffer,	fk422	0.0	1.9	3.0	2.7	2.3	2.0	0.8	1.8	3.2	2.7
oxidation”	10 mM MgSO4, dATP concentration	fk277	0.0	0.0	0.0	0.0	0.0	0.0	0.0	0.0	0.0	2.7
Table S1, S2												
Bst DNA Polymerase, Large fragment, 37 deg,		fk380	0.0	0.2	0.3	0.1	0.1	0.2	0.1	0.1	20.9	9.5
ThermoPol® Reaction Buffer , 10 mM MgSO4, dATP		fk381	0.0	0.1	0.2	0.2	0.1	0.2	0.1	0.1	23.5	10.9
"no-	No single extension step	fk457	0.0	0.0	0.1	0.1	0.1	0.1	0.1	0.1	1.0	0.7
extension"		fk458	0.0	0.0	0.1	0.1	0.2	0.1	0.1	0.1	1.0	0.7
Klenow Large Fragment (3'→5' exo), 37 deg,		fk419	0.0	4.7	6.3	3.8	2.5	3.9	4.9	3.7	41.2	35.2
ThermoPol® Reaction Buffer I, 10 mM MgSO4, dATP		fk420	0.0	7.1	9.0	5.2	3.2	4.1	2.5	4.4	47.6	42.8
Deep Vent® (exo-) DNA Polymerase , 37 deg,		fk423	0.0	1.1	1.8	1.2	1.0	0.9	0.5	0.9	35.0	33.3
ThermoPol® Reaction Buffer I, 10 mM MgSO4, dATP		fk424	0.0	0.6	1.2	1.0	0.9	0.8	0.5	0.7	31.2	29.5
Sulfolobus DNA Polymerase IV , 37 deg,		fk459	0.0	0.0	0.0	0.1	0.1	0.1	0.1	0.1	0.9	1.0
ThermoPol® Reaction Buffer I, 10 mM MgSO4, dATP		fk460	0.0	0.3	0.7	0.1	0.4	0.5	0.3	0.3	5.6	3.5
